# Supplementary material for: Transcriptomic Analysis Reveals the Regulatory Mechanism of Cold Tolerance in Saussurea involucrata: The Gene Expression and Function Characterization of Dehydrins
Source: Int J Mol Sci. 2025 Sep 17;26(18):9030. doi: 10.3390/ijms26189030 (PMC12470158; doi:10.3390/ijms26189030)
Supplement: Supplementary file 1 [file ijms-26-09030-s001.zip › ijms-3852665-supplementary.pdf]

Table S1 The quantitative PCR primers of putative genes.

No.	Gene id	Forward Primer (5'→3')	Reverse Primer (5'→3')
1	CL227.Contig8	GAGGAGCCACAGAGTCGT	CCGATACCTCCATGCTGA
2	CL227.Contig10	ATACCACGCCGACCTGAA	CTCCTTGCCCATCATCCT
3	CL227.Contig14	ATATGGAGGAGATAAGCAGTA	GGCATGACCCACTTTTGTA
4	CL1767.Contig3	GAAGAGGGCTCAGATGGA	GTTGTCGTCCTCAAGTGTC
5	CL1854.Contig6	TGCGGAGGAGAACAACAC	ACTGCCACTCCCAGAACG
6	CL1854.Contig7	GCAGCCTTGTGCTCATCG	AACCCACTTCAGCCCACC
7	CL9026.Contig1	GTCCACAATCCACTTCAG	TCACTCCCTTCTTCTCAT
8	CL9026.Contig5	ATGTCCACAATCCACTTC	TTCTCCATCACTCCCTTC
9	CL9026.Contig11	GGTGGTGTTGGTGGCGGT	GGGAAAGGTGTGGTGGGG
10	CL9026.Contig12	AAGGTGGGAAAGGTGTGG	CGGTGGTGTTGGTGGCGG

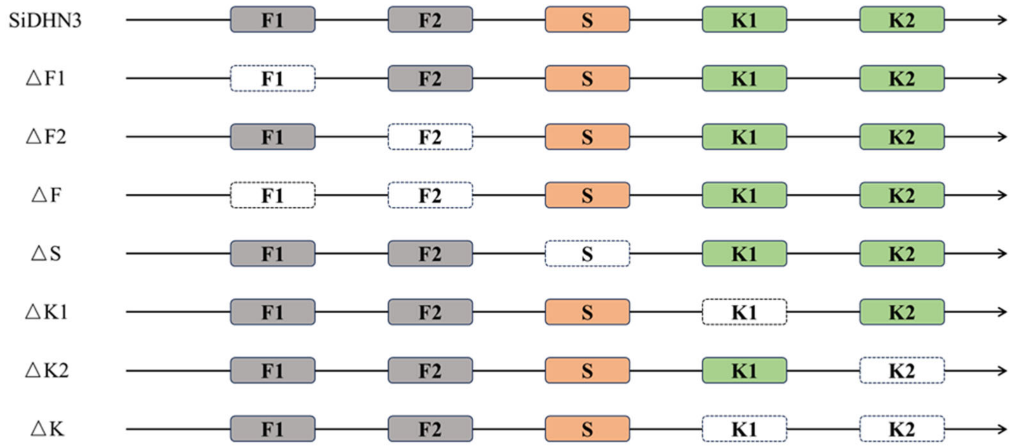

Figure S1 Schematic representation of the diverse segments of SiDHN3. positions of the conserved segments F (gray box), S (orange box), and K (green box). The dotted box indicates the deleted sequences.

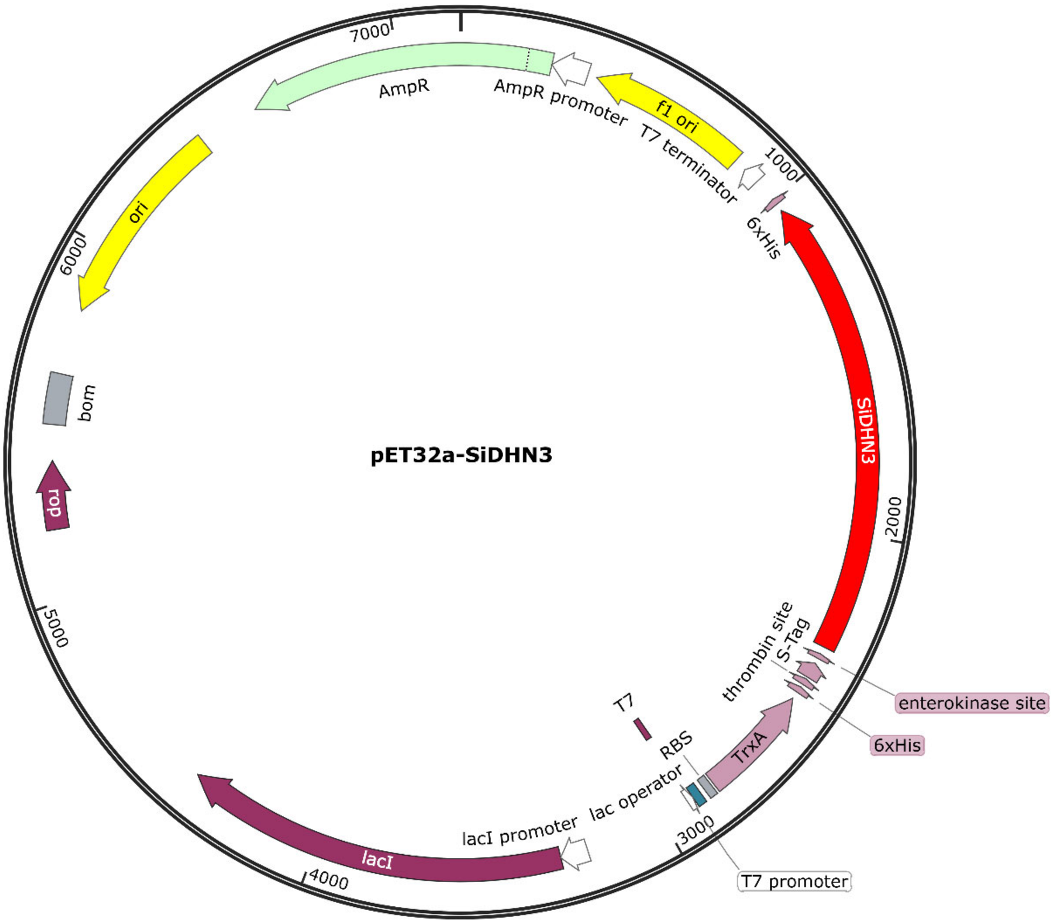

Figure S2 Schematic diagram of the pET32a-*SiDHN3* plasmid
